# Dance, Music, Meter and Groove: A Forgotten Partnership

**DOI:** 10.3389/fnhum.2016.00064

**Published:** 2016-03-01

**Authors:** W. Tecumseh Fitch

**Affiliations:** Department of Cognitive Biology, University of ViennaVienna, Austria

**Keywords:** dance, music, rhythm, meter, groove, musicality

## Abstract

I argue that core aspects of musical rhythm, especially “groove” and syncopation, can only be fully understood in the context of their origins in the participatory social experience of dance. Musical meter is first considered in the context of bodily movement. I then offer an interpretation of the pervasive but somewhat puzzling phenomenon of syncopation in terms of acoustic emphasis on certain offbeat components of the accompanying dance style. The reasons for the historical tendency of many musical styles to divorce themselves from their dance-based roots are also briefly considered. To the extent that musical rhythms only make sense in the context of bodily movement, researchers interested in ecologically valid approaches to music cognition should make a more concerted effort to extend their analyses to dance, particularly if we hope to understand the cognitive constraints underlying rhythmic aspects of music like meter and groove.

## Introduction

When I began studying African drumming with master Ghanaian drummer Martin Obeng, he continually emphasized that it is not sufficient to correctly play the bell or drum part: performers must also be able to dance appropriately to the music while playing their instruments (and often, singing). By this Obeng did not intend us to engage in any fancy dance moves: a simple back-and-forth stepping, with small leg and hip movements, was enough. But one had to feel and subtly express the basic underlying pulse, clearly and in the correct temporal location, to properly understand how the complex and polyrhythmic layers of (for example) *agbekor* fit together and cohere. Despite the initial challenge of playing the complex and syncopated bell pattern while moving, I soon found that the movement not only became effortless, but also provided a crucial and semi-automatic metrical framework aiding my perception of this music. Today I cannot hear the *agbekor* bell pattern without (at least covertly) sensing this underlying pulse. I had a similar experience when I began singing and playing percussion in a large salsa band, where we singers were expected to continually dance, and sometimes perform more complex twirls and moves, in time and in synchrony with each other. Again this was initially a challenge for someone whose previous performance experience involved singing in a church choir, or playing guitar in folk and rock styles, but with practice the movement became second nature. And again, I can’t hear a salsa *clave* or bass line today without immediately and automatically feeling and (at least implicitly) moving to that essential pulse.

The core thesis of this essay is that such rhythmic movement is a central but often neglected factor in a very wide variety of musical genres, especially dance music, but also including musical styles like jazz and some classical music, for which it is no longer customary to dance. I will argue that important rhythmic aspects of such music, especially meter and syncopation, cannot be properly understood without reference to movement and dance, and that the persistent tendency of “art music” to divorce itself from motion and dance is a regrettable phenomenon to be resisted by both audiences and theorists. Although the main examples I will explore here concern the relatively recent styles of jazz and salsa, I believe that much of what I say would be equally applicable to the gigues and gavottes of Bach or minuets of Mozart, whose essential connection to the baroque dances for which they are named was lost much longer ago. The more recent process by which jazz was “concertized” is better documented but not, I think, different in kind.

**Figure 1:**
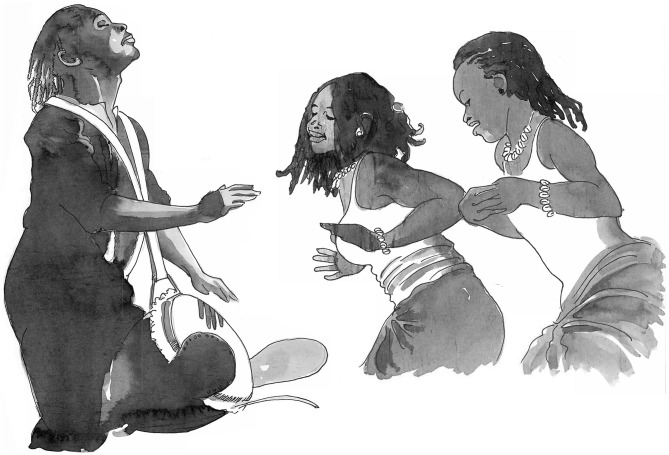
**Dance and drumming**. The painting illustrates how, in many musical traditions, music (here, djembe drumming) and dance are equally important components of a participatory social music experience (painting by the author).

Thus my purpose here will be to draw out the implications of elevating human dancing to the status of equal partner in rhythmic music and as an unsung hero in the origins of meter and rhythmic cognition in our species. Figure 1 illustrates this motif visually in a participatory African drumming context, where dancing is typical, and shared improvisation and back-and-forth between dancers (who may also sing) and drummers is commonplace (cf. Bebey, 1975; Locke, 1982, 1996). Increased attention to entrained bodily movements is particularly opportune now that we know that a handful of nonhuman species also possess an ability to entrain their bodily movements to a musical beat (cf. Ravignani et al., 2014; Fitch, 2015a), thus broadening the range of comparative and neurobiological techniques available for research on rhythmic cognition. From an experimental viewpoint, movements made to music often provide the main behavioral readout of cognitive processing of rhythm and “groove” (Madison, 2006; Janata et al., 2012; Merker, 2014), and thus underlie practical empirical methods for testing hypotheses about rhythmic cognition in our own and other species (e.g., Patel et al., 2009; Cook et al., 2013). Thus I argue that musical movement provides a crucial and often overlooked common denominator for understanding rhythm.

## Movement and The Biology of Rhythm

Treating movement and dance as key components of rhythmic music makes good biological sense from both neural and ecological viewpoints (Fitch, 2015b). In terms of neurobiology, one of the most robust findings in the neuroscience of music is that attending to rhythmic aspects of music activates motor areas of the brain (e.g., Chen et al., 2006; Grahn and Brett, 2007; Grahn, 2012; Merchant et al., 2015), even in the absence of any overt movement. Body movement also strongly modulates rhythmic perception, particularly affecting metrical interpretation (Phillips-Silver and Trainor, 2005, 2007). It thus appears that motor regions provide part of the necessary predictive scaffolding that enables the auditory system to properly “parse” a musical surface into an appropriately structured rhythmic representation (Schubotz, 2007). Although the difficulties of doing brain imaging during actual dancing are formidable, a PET study in which participants performed small tango dance movements with their feet on a board, and where muscle contraction, auditory stimulation and other factors were controlled, yielded results clearly consistent with this hypothesis (Brown et al., 2006). Because the neural bases of rhythmic entrainment have recently been reviewed in detail (see Merchant et al., 2015), I will not discuss them further here, but instead delve into more ecological and formal issues.

From a broad, ecologically valid perspective on human musical practice, it is clear that in most cultures music is often, or even typically, accompanied by some form of dance (by which I simply mean appropriate body movements *not* required to actually produce the music). In such cases, there is an intimate relation between musical signals and bodily movements that makes them, culturally speaking, part of a single greater whole (cf. McNeill, 1995; Fitch, 2015b). In many genres, performing the dance without the proper music would be bizarre, and performing the music without the dance seems incomplete and unsatisfying (in my days playing in a salsa band, there was nothing more frustrating than an audience that stayed seated and clapped politely at the end of each number—salsa is *dance music*). The connection between music and dance is so close that many languages do not even distinguish the two, but use a single word to connote both (Merker, 1999; Nettl, 2000), as indeed did the Greek word “mousike” that is the etymological ancestor of our English “music.” Similarly, if one examines the wide variety of definitions of the term “rhythm” one sees that, at its Greek beginning (e.g., for Plato) movement was the crucial concept, but the centrality of movement has gradually been bleached away into purely auditory and cognitive interpretations today (see the list of definitions presented on pages 2–3 of Toussaint, 2013). Given the still-pervasive link between music and dance in the most popular styles today and its anthropologically well-documented omnipresence in traditional societies, I find speculations that dance and “moving together in time” were central aspects of the evolution of music (cf. McNeill, 1995) quite plausible.

## Meter and Movement

Almost any motion intended to be repeated indefinitely will have some form of cyclicity: minimally requiring “go out” and “come back” components that allow the motion to return to its starting point in order to begin anew. Thus even the simplest regular tapping of a foot inevitably has an up and a down component. Bipedal walking adds an additional twist: the alternation of right and left leg, the two of which make up a complete cycle. If one wishes to keep count of how many steps one has walked, the first required decision is whether to count paces (so that each footfall is counted) or to count cadences or cycles (so that each right-left combination is counted once). While on its face this is an arbitrary decision, with no “correct” answer, there are many situations where it is more natural to count cycles (e.g., for a man with a limp, a child skipping, or for a quadruped’s gallop). Thus, the simplest possible 2/2 meter derives, quite naturally, from attending to simple bipedal locomotion. But given that we must both raise and lower each leg during bipedal walking, the cycle-counting convention leads to a four-way subdivision of each cycle: right – up – left – up. This yields a 4/4 meter, with the 1 and 3 as “downbeats” (where the foot makes contact) and the 2 and 4 as upbeats (where one foot is maximally raised). Thus we need look no further than a man walking down the street to witness two of the most basic and pervasive musical meters expressed in movement, and indeed some of the most popular and infectious dance styles, including reggae, merengue or (street) samba can be danced to by simply walking in place.

Three-way divisions also fall naturally from slightly more varied movements. For example, in the Viennese waltz, turns can be interspersed with steps (step – turn – feet together), where each “together” provides a preparatory pause for the next step with the opposite foot. This yields an overall six-way cycle for both right and left foot to step, or in a simple box waltz with four half-turns, a 12-part cycle to return to the starting place. Although there are many ways to dance the waltz, all make use of this basic alternation between movement and pausing to yield groups of three rather than two or four, again leading naturally to the 3:4 waltz meter. Like many dances, the waltz originated as a country dance and was initially viewed as scandalously indecent by aristocrats due to the characteristic close, almost hugging, position of the male and female dancer (Stephenson and Iaccarino, 1980). But once established among polite society in 18th century Vienna, it proved so infectious as to spread around the world, bringing its 3:4 meter along with it.

Another very popular family of dance styles, loosely termed “swing” dancing, illustrates an interesting variant on this theme. Swing dance has its origins in African-American jazz dances like the “hop”, which was renamed the “Lindy hop” after Charles Lindbergh’s solo “hop” across the Atlantic in 1927 (Gioia, 1997; Crease, 2000). The basic swing style alternates between a “closed” position, with partners joining both hands, and a breakaway “open” phase, in which the partners are joined by only one hand, during which various improvisatory swings and moves can occur (Stephenson and Iaccarino, 1980). The dance thus alternates between European and more African partner positions with each cycle. But unlike the waltz, and true to its African roots, the six-count of the dance step (long-long-short-short) contrasts with a 4:4 musical rhythm to yield an exciting polyrhythmic tension between the dancers and the music. In some swing styles, this goes even further and the “open” phase switches to an eight-count, while the closed unfolds to a six-count. In either case, swing dancers phase in an out of metrical register with the music to yield a much longer combined cyclicity than that of either the music or dance alone.

Echoing the waltz, swing was a dance with African-American folk origins that exploded in popularity among both blacks and whites and particularly during and after World War II, was embraced and danced in nations around the world. Swing dance was intimately bound up in the origins of jazz music (Gioia, 1997; Crease, 2000), and the eventual divorce of jazz music from dancing provides a rich and recent illustration of how music’s rhythmic origins in dance can be submerged or even erased over time (to which I return below).

To summarize, musical meters from the simple to quite complex can be closely linked to particular modes of human bodily movement. It is somewhat remarkable that groups of two and three, combined multiplicatively or additively, provide sufficient resources to yield the diversity of metrical structures typifying virtually all human music. This seems less surprising if we hypothesize that musical rhythms inevitably make reference to movements of a few simple sorts (perhaps some additional meters might feel natural to quadrupedal mammals, six-legged insects or eight-legged spiders). Indeed, all of the rhythms I will discuss in the rest of this article follow naturally from of the most basic 4:4 meter already implicit in simple bipedal walking.

## What Makes Some Rhythms Groove?

A meter provides a very general framework within which an unlimited diversity of specific rhythmic patterns can be generated. But, as recognized since ancient times (Toussaint, 2013), not all rhythms are equally exciting or propulsive to dancers. Given a particular meter, what makes certain rhythms “groove” and others fall flat? My argument in this section will be that this issue, too, can only be understood by relating it to the movements one makes with the music, and that a rhythm that would “groove” for one dance pattern might fall flat for another.

To be clear, I start by distinguishing “swing” from “groove” (cf. Honing and de Haas, 2008; Merker, 2014). A fundamental aspect of most dance music is its isochronicity, whereby a regular periodic pulse or “beat” is established to which the dancers can synchronize their periodic movements. This pulse (a.k.a. “tactus”) provides a framework upon which expressive deviations may be superimposed, such that the musical surface is typically not precisely periodic, and different musical styles allow different degrees of expressive deviations from the pulse. It is sometimes supposed that it is such deviations in micro-timing that makes music “groove” or “swing”. However, there seems to be a tradeoff between expressive timing and general rhythmic complexity, such that polyrhythmic and syncopated rhythmic styles allow little deviation from strict periodicity, while relatively simple and repetitive rhythms are more open to expressive deviation from the pulse (Temperley, 2004). Furthermore, most dance music is in fact rather strictly isochronic, and nonetheless has a very strong and compelling groove. Thus I follow Merker (2014) in seeing rhythmic deviations from strict periodicity as orthogonal to the issue of groove, and I interpret the term “groove” to indicate whatever factors make a particular rhythm conducive to movement in listeners, even when played in strictly isochronous manner (e.g., by a drum machine). I reserve “swing” to indicate that subset of the class of expressive deviations from isochrony (mostly in jazz) that, by being relatively predictable, remain compatible with dancing. These are fundamentally different concepts, and I will discuss only groove here.

One of the core mysteries in understanding the rhythmic underpinnings of many modern dance styles is their pervasive use of syncopation (Temperley, 1999, 2004). Syncopation occurs when the local temporal expectations generated by a particular meter are violated by placing notes at “weak” locations, normally unaccented, while omitting them at the expected “strong” locations (Longuet-Higgins, 1979; Temperley, 1999; London, 2004; Fitch and Rosenfeld, 2007). Empirical work on syncopation indicates that syncopated rhythms are more difficult to play and less memorable, and that too much syncopation drives listeners to reevaluate the meter, shifting the inferred pulse phase to yield a less-syncopated rhythmic interpretation (Patel et al., 2005; Fitch and Rosenfeld, 2007). One might expect, given these perceptual/cognitive liabilities, that syncopated rhythms would be strongly dis-preferred in music designed to establish and maintain an accessible and consistent pulse. This makes the pervasive use of syncopation in many styles of dance music a central puzzle in rhythmic cognition.

## Syncopation: Energizing The Upbeat

I hypothesize that this mystery can be readily solved if we enlarge the scope of inquiry to incorporate movement and dance. This solution is directly implied by the musical distinction between “downbeat” and “upbeat”. The first prerequisite of a grooving rhythm is that it establishes a clear and consistent meter, where both the pulse (defining the tempo of movement) and meter (which will determine where the dancers initiate and terminate their periodic movement cycles) are evident to the listeners (Fitch, 2015a). This will result in footfalls synchronized to the downbeat.

However, successful syncopated rhythms add acoustic energy on upbeats, when the legs (or limbs, or whole body) are moving upward, between the downbeats. More technically, by adding acoustic energy to phases in the movement cycle where movements are directed away from gravity, syncopation makes the dancer feel these upward movement components as coherent with the musical surface. The net effect is that syncopations essentially “inject energy” into upbeats, adding an exciting, propulsive, and light “feel” to the rhythm that is absent in a “straight” rhythm that simply emphasizes the downbeats, when the feet strike the floor. Thus, for a suitably experienced dancer, more syncopated rhythms feel exciting and energizing in comparison with a straight “four on the floor” rhythm that feels flat and plodding because it emphasizes only downbeat energy (particularly with the low frequency energy pulses generated by the bass and kick drum, which play a disproportionate role in determining the pulse and meter; Hove et al., 2014).

**Figure 2:**
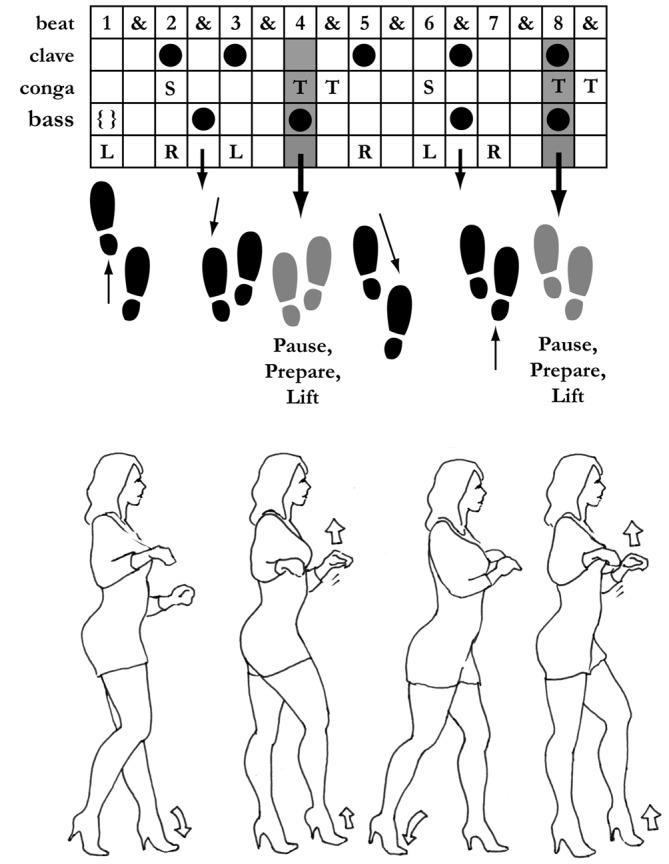
**Salsa dance and syncopation**. The diagram illustrates a common version of the basic salsa step (“dancing on one”), synchronized with the instrumental parts to illustrate how a syncopated bass event (on the four) occurs during the upwardly-directed preparatory portion of the step, injecting acoustic energy into this upbeat (figure by the author). Downward arrows indicate syncopated bass notes; the { } indicates the absent bass (a rest) on the “one” downbeat.

What does it mean for a dancer to be “suitably experienced” to deal with syncopated rhythms? Familiarity with the overall musical style and its conventions must play a crucial role because the perception of meter depends not only on “bottom up” perceptual factors, but also on “top down” expectations generated by the listener (London, 2002, 2004). To take a prominent example, contemporary salsa music makes use of a strongly and consistently syncopated bass pattern, known as the *tresillo* or *tumbao*, where the bass plays the offbeat eighth note just before the “three” downbeat, and plays nothing on the “one” (Mauleón, 1993; Moore, 2010). This anticipatory offbeat typically coincides with, and is presumably originally derived from, a low open tone on the conga drum (a distinct pattern that is also known as *tumbao*), yielding a strongly syncopated musical surface. However, in one common standard salsa dance step, illustrated in Figure 2, this syncopation is precisely the moment during which a pause in the repeated 4/4 stepping pattern is open to improvisational flourishes, whether a small kick or a full twirl. The result, for the dancer, is a strong rhythmic acoustic emphasis at the points where the “sauce” of salsa dancing is most clearly manifested. This is in sharp contrast to some other popular Afro-Caribbean rhythms, such as *merengue*, which have a much clearer and unsyncopated bass line, and are thus much easier for beginners to dance too (Moore, 2010).

To those unfamiliar with salsa music, this concentration of low-frequency energy on offbeats makes it a challenge to even *locate* the downbeat cognitively (although other instruments, including both vocals and percussion, make the meter clear nonetheless). But to an experienced salsa listener/dancer, the context of the other instruments (particularly the *clave*, a percussive ostinato at the heart of salsa rhythms) makes the meter obvious, and the injection of bass energy at the syncopated upbeat point in the dance step is precisely what makes salsa groove. Indeed, the step I have discussed here and illustrated in Figure 2 is only the simplest and most straightforward variant of salsa (sometimes termed “dancing on the one”). In a more complex and arguably more “authentic” variant, this entire pattern is delayed by one quarter note (“dancing on the two”), leading to a closer fit between the dancer and the *clave* and an even greater feeling of fluid but syncopated energy in the dance. In this case, the dancer now steps in time with the “offbeat” bass and performs the “up” portion of the dance on the one—an even greater degree of syncopation than in the illustrated version (which itself can be a challenge for many beginning salsa dancers).

Thus, I suggest that syncopations—on their face violations of meter that should create cognitive dissonance—make perfect sense from the viewpoint of a dancing listener. The dancers’ own movements are manifesting the downbeat clearly enough to need no particular reinforcement from the musical surface (or at least from the low-frequency bass and drum parts). Instead, the music is free to add offbeat energy at various other points in the “locomotory” cycle of steps, for example on upbeats to make the music feel lighter, more upwardly directed (and indeed more “upbeat” in the metaphoric sense). Looked at from a purely musical, cognitive viewpoint, and divorced from movement, syncopation seems almost perverse, and the popular success of syncopated dance styles appears mysterious. But viewed from the cognitive perspective of the dance floor, I suggest that syncopations of various types can be very naturally interpreted as emphases of components of the dance other than the plodding downbeat.

In his sweeping critiques of jazz, famed critic Theodor Adorno thoroughly misses this point by lambasting the way jazz music embraces the “ostensibly disruptive principle of syncopation, yet without ever really disturbing the crude unity of the basic rhythm, the identically sustained metre, the quarter note”. He goes on to ridicule jazz listeners’ (and dancers’) evident capacity to be “impervious to disruptive factors like syncopations” as showing nothing more than a clever “ability to cope with obstacles” (Adorno, 1968). One can readily imagine that Adorno never personally experienced the contagious energy and excitement of being part of a roomful of swing dancers doing the Lindy hop.

I conclude that one of the central factors underlying groove—the capacity of certain types of music to compel movement—is its tendency to add energy on offbeats that coincide with particular movements in the dance, particularly upbeats, and thus to add “disruptive” syncopations while still conveying a clear meter.

## Dance and Music Go Their Separate Ways

If we accept the premise that many forms of music find their rhythmic and metrical origins in a symbiotic relationship to corresponding dance styles, we are led to ask why musical genres and dance styles often show a historical tendency to diverge from one another. While millions of listeners around the world still delight in Bach’s French Suites, it is unlikely that more than one in a thousand would know how to dance a gigue, gavotte or sarabande. Even jazz, a genre whose origins as dance music are historically undeniable (Crease, 2000), is more likely today to be enjoyed by quietly seated listeners than by a roomful of dancers. Even Latin jazz, with its roots in Afro-Cuban dance rhythms like son, rhumba and guaguanco that are also the forerunners of salsa (Moore, 2010), today is often enjoyed at concerts from a seated position (though I have witnessed some rather energetic “dancing” occur in those seats …). If music and dance styles are typically intimately linked in their origins, how can we explain why such apparently frequent “concertization” of music occurs?

I suggest that the origins of this phenomenon are to be found in the pervasive tendency in Western culture to distinguish “high art” from folk styles and “mere entertainment”. This dichotomy encourages an attitude whereby, for a musical form to be taken seriously, it must “rise above” the fun-loving, participatory environment in which dancing plays a central role. This seems particularly true of those styles whose dance originates among the musically-unsophisticated “lower” classes, as did so many of today’s most popular dance styles (waltz, swing, calypso, reggae, salsa). For the music associated with such styles to qualify as “serious music”, among those adopting this attitude, it seems it must be concertized and thus divorced from such lowly origins.

Jazz provides a very clear and well-documented example of this phenomenon. From its ragtime origins through to the swing era, jazz was closely associated with dancing (Southern, 1971; Gioia, 1997). Swing dancing capitalized on a newly respectable dancehall phenomenon that started with the foxtrot to rapidly become both the musical and dance style of the entire USA (Stephenson and Iaccarino, 1980; Crease, 2000). But there were moves to separate jazz music from dancing quite early on. In 1924, Paul Whiteman staged a jazz concert in New York’s Aeolian Hall that culminated in a performance of George Gershwin’s *Rhapsody in Blue*, with the composer himself at the piano. This concert was intended to show that jazz had risen above its southern black roots and was ready to ascend to the level of serious, educated urban music (Crease, 2000).

By the 1930’s, two distinct jazz subcultures, dubbed the “alligators” and the “jitterbugs,” had formed. While “jitterbugs” danced (often in extreme and outrageous manners), the “alligators” crowded to the front of the bandstand and simply listened reverently (and often needed to be dispersed by bouncers). In 1937, *Down Beat* magazine (then and now the preeminent venue for “alligators” turned jazz critics) published an article titled “Don’t Spit on the Jitterbug—Educate Him!” Nonetheless, swing jazz remained predominantly dance music through the second World War, when for a brief moment its popularity extended around the planet. But this divorce between “art music” and “dance music” took its time happening: when Benny Goodman played at Carnegie Hall, the teenagers in the audience reportedly danced in the aisles (Gioia, 1997, p.125).

With the rise of bebop and the birth of rock-and-roll (to which the Lindy hop was readily adapted as the “jitterbug”; Gioia, 1997), the swing era came to an end. Jazz rapidly ceased to be considered dance music (although Dizzie Gillespie, one of the kings of bebop, famously remarked that he could “dance his ass off” to bop, and that “jazz should be danceable” and “make you wanna move”). By 1948, with dancehalls around the nation closing, and the primary income for most jazz musicians drying up, *Down Beat* could change its tune to editorialize that “jitterbugs were a precious nuisance, but brother, we certainly could use them.” Thus, the future of jazz lay ultimately with the alligators.

It seems clear that one can *enjoy* any form of music without actively dancing to it, including those like waltz, jazz or salsa that have their origins as dance music. Like most Bach fans, I certainly enjoy his French Suites, and many other classic works, without knowing how to dance a gigue. Nonetheless, I had listened to jazz for many years before learning to dance swing, and I certainly feel that this experience enriched my cognitive experience of this style (even in its more rarified forms, like Gershwin or Miles Davis). Similarly, until I learned to dance salsa (in a one-room shack in the middle of a Puerto Rican sugarcane field, incidentally), I truly had no concept of what salsa music was about. Perhaps my experience of Bach would be similarly enriched by learning to dance the sarabande.

At a minimum, if we want to understand the rhythmic *origins* of a musical style, it behooves us to know how contemporaries would have moved to that music. In some cases, this might help make sense of such pervasive but otherwise puzzling phenomena as syncopation. But more generally and sadly, I feel that the concertization of music tends to divorce it from its *biological* roots as a vital participatory social phenomenon (cf. Fitch, 2015b). Thus, in the same way that modern researchers extol the (unconscious) cognitive sophistication of “non-musician” listeners (Honing, 2009), and tend to downplay the historically emphasized distinction between them and their educated, musically literate brethren, I suggest that broadening the scope of music cognition research to include dance (especially popular improvisational dance, rather than the choreographed dance routines of professional dancers) might offer new and significant insights into the nature and origin of musical rhythm.

## Author Contributions

The author confirms being the sole contributor of this work and approved it for publication.

## Funding

Supported by ERC Advanced Grant SOMACCA (#230604).

## Conflict of Interest Statement

The author declares that the research was conducted in the absence of any commercial or financial relationships that could be construed as a potential conflict of interest.
